# Multi-omics analysis reveals the mechanism of selenite reduction by *Rhodococcus qingshengii* strain isolated from selenium-rich mine

**DOI:** 10.3389/fmicb.2025.1712891

**Published:** 2025-11-14

**Authors:** Jianhui An, Dandan Yi, Jing’e Wu, Guang’ai Deng, Zhiyong Wang, Mu Peng

**Affiliations:** 1 Hubei Key Laboratory of Biological Resources Protection and Utilization, Hubei Minzu University, Enshi, China; 2 College of Biological and Food Engineering, Hubei Minzu University, Enshi, China

**Keywords:** *Rhodococcus qingshengii*, selenite reduction, transcriptomics, metabolomics, detoxification

## Abstract

*Rhodococcus* species are renowned for their metabolic diversity and environmental adaptability, yet their selenium metabolism remains insufficiently studied. In our previously work, we isolated a highly selenite-tolerant strain, *Rhodococcus qingshengii* PM1, from selenium-rich soils in Enshi, China. To reveal the reduction mechanism of sodium selenite, integrated transcriptomic and metabolomic analyses were conducted. Biochemical assays confirmed that Se exposure induced pronounced oxidative stress in strain PM1 and elicited strong induction of the antioxidant defenses. A total of 308 differential metabolites were detected, with bioactive compounds, organic acids, lipids, secondary metabolites and organoheterocyclic compounds. A total of 1,511 differentially expressed genes were identified. These changes were primarily associated with sulfite reductase complex genes (*CysNDHIJ*), Fe–S cluster biosynthesis genes (*SufBCDSE*), glutathione metabolism, lipid remodeling, redox metabolic pathways and antioxidant pathways, all contributing to the detoxification and reduction of selenite. Notably, metabolites such as prostaglandin D3 were upregulated, reflecting lipid signaling in response to selenium, while others including physangulide, enhydrin, and sebacic acid were downregulated, indicating a metabolic shift away from lipid biosynthesis and secondary metabolism. These findings elucidate the molecular mechanisms underlying microbial selenite detoxification and highlight *R. qingshengii* PM1 as a promising candidate for bioremediation of selenium-contaminated environments.

## 1 Background

Selenium (Se) is an essential trace element involved in various biological processes. It not only significantly influences plant growth, development, and stress responses (Abdullah et al., 2025), but also plays a critical role in the prevention and treatment of human diseases such as cardiovascular disorders, inflammation, and cancer (Chen et al., 2025). Selenium exists in multiple chemical forms in nature, including seleno-amino acids, selenoproteins, selenates, selenides, and selenite (Gu and Gao, 2022). However, the safe intake range of selenium is narrow, with a small margin between the required dose and toxic levels (Kieliszek and Błażejak, 2013; Dominique et al., 2023; Kieliszek and Serrano Sandoval, 2023). The United States Department of Agriculture (USDA) recommends a daily intake of 55 μg for adults, while the European Food Safety Authority (EFSA) suggests 60–70 μg/day; intake above 400 μg/day is considered excessive, and levels reaching 800 μg/day may lead to toxicity (D'Amato et al., 2020; Schiavon et al., 2020). Both natural geological processes (e.g., weathering, volcanic activity, and sediment leakage) and anthropogenic activities such as mining, smelting, cement production, and coal combustion contribute to the continuous accumulation of inorganic selenium in soils and water bodies, exacerbating environmental contamination (Sinharoy et al., 2019; Genchi et al., 2023). To address the increasing problem of selenium pollution, various removal methods have been developed, including physical techniques (e.g., membrane filtration, volatilization, and adsorption), chemical treatments (e.g., precipitation and reduction), and biological approaches (Yang et al., 2025). Among these, microbial reduction of highly soluble and toxic oxidized selenium species (such as SeO₄^2−^ and SeO₃^2−^) into low-toxicity and poorly soluble elemental selenium (Se⁰) represents a practical and promising bioremediation strategy (Ullah et al., 2023).

To date, various bacteria, archaea, and fungi have been identified that can efficiently detoxify selenite (SeO₃^2−^) into elemental selenium (Se⁰) and synthesize selenium nanoparticles (SeNPs) under both aerobic and anaerobic conditions (Xue et al., 2024). The microbial mechanisms of heavy metal detoxification have been extensively characterized in multiple studies (Alotaibi et al., 2021). Through long-term evolution, bacteria have developed diverse defense systems to protect cells from selenite-induced oxidative toxicity (Painter, 1941; Zhang et al., 2020). These detoxification mechanisms are mainly classified into non-enzymatic and enzymatic pathways: in the non-enzymatic pathway, bacteria reduce selenite mediated by endogenous reductants such as glutathione, hydrogen sulfide, and iron carriers (Nancharaiah and Lens, 2015). In the enzymatic pathway, under anaerobic conditions, enzymes including fumarate reductase (FccA) (Li et al., 2014), nitrite reductase (Nir), hydrogenase I, and arsenate reductase catalyze the reduction of selenite to elemental selenium (Se⁰), whereas under aerobic conditions, this process is jointly facilitated by thioredoxin reductase (TrxR) and sulfite reductase (CysJI) (Nie et al., 2023; Ge et al., 2024). In addition, oxidative stress response, DNA damage repair, and Se(IV) efflux also play important roles in bacterial tolerance to heavy metal stress (Pinel-Cabello et al., 2021a,b). Although numerous studies have elucidated microbial mechanisms of selenite detoxification, efficient and sustainable remediation strategies for selenium pollution remain to be further explored.

The genus *Rhodococcus* comprises Gram-positive actinobacteria with high G + C content, widely distributed in soils, aquatic environments, and various contaminated sites, exhibiting remarkable metabolic diversity and environmental adaptability (Nazari et al., 2022). Members of this genus are capable of degrading a variety of organic pollutants, including petroleum hydrocarbons (D’Ugo et al., 2025), polycyclic aromatic hydrocarbons (PAHs) (Ma et al., 2023), phenolic compounds (Barik et al., 2021), and dyes (Maniyam et al., 2020), and also demonstrate strong tolerance to and transformation ability of heavy metals (Goswami et al., 2017). The bioremediation potential of *Rhodococcus* strains is closely linked to their unique cellular structure and enzymatic systems, such as cell walls rich in mycolic acids that enhance tolerance to ecological toxins, as well as a variety of specialized metabolic enzymes (Kuyukina and Ivshina, 2019; Blumenstein et al., 2022). Although *Rhodococcus* exhibits substantial heavy metal reduction capacity and can withstand toxic substances and harsh environments (Adenan et al., 2020; Zhao et al., 2021; Hu et al., 2025), its role in selenium pollution transformation and remediation remains underexplored and lacks systematic investigation (Presentato et al., 2018; Morris et al., 2024).

This study focuses on *Rhodococcus qingshengii* PM1, isolated from a selenium-rich mine in Enshi City, which exhibits significant selenium-reducing capability (Wang et al., 2025). By integrating transcriptomic and untargeted metabolomic analyses, we comprehensively investigated the response mechanisms of PM1 under selenite stress. The results not only reveal the adaptive strategies of PM1 but also provide a theoretical basis for understanding microbial tolerance to selenite at the molecular level. This research offers valuable insights for advancing environmental remediation and bioremediation technologies.

## 2 Materials and methods

### 2.1 Overview of strain PM1 and growth culture

The selenite-reducing strain *R. qingshengii* PM1 was previously isolated from selenium-rich carbonaceous mudstone soils collected in Yutangba, Enshi, Hubei Province, China. A series of physiological, biochemical, and phylogenetic analyses confirmed its taxonomic identity and strong selenite reduction ability, including tolerance to up to 100 mM Na_2_SeO_3_ (Wang et al., 2025). The strain has the unique ability to reduce selenite to elemental selenium, which subsequently forms selenium nanorods (SeNRs). The complete genome sequence has been deposited in GenBank under accession number CP104782. For this study, strain PM1 was cultured in LB medium supplemented with or without 50 mM Na_2_SeO_3_ under the following conditions: the culture was grown at 28 °C with 160 rpm shaking and incubated until reaching the logarithmic growth phase, approximately 15 h. This study further investigated the transcriptomic and metabolomic response mechanisms of strain PM1 under selenite stress.

### 2.2 Measurement of key enzyme activities

To evaluate the oxidative stress response of strain PM1 under selenium treatment, the activities of hydrogen peroxide (H_2_O_2_) and malondialdehyde (MDA), superoxide dismutase (SOD), peroxidase (POD), ascorbate peroxidase (APX), glutathione S-transferase (GST), glutathione reductase (GR), and thioredoxin reductase (TrxR) were measured using standard enzyme assay kits from Sangon Biotech Co., Ltd. (Shanghai, China) with the following catalog numbers: D799773-0050, D799761-0050, D799593-0050, D799591-0050, D799597-0050, D799461-0050, D799611-0050, D799261-0050 and D799259-0050. Two treatments were applied: a control group and 50 mM Na_2_SeO_3_ treatment group. Cells from both groups were collected after cultivation and lysed in ice-cold extraction PBS buffer. The enzyme activities were then determined according to the manufacturer’s instructions using appropriate enzyme activity kits or standard methods. The assay conditions for each enzyme were as follows: H_2_O_2_ was quantified using a horseradish peroxidase–coupled colorimetric assay, and MDA was measured by the thiobarbituric acid reactive substances (TBARS) method. SOD activity was determined by measuring the inhibition of the chemical reaction induced by superoxide radicals. POD activity was measured based on the decomposition of hydrogen peroxide. CAT activity was quantified by monitoring the rate of hydrogen peroxide degradation. APX activity was measured by the reduction of ascorbate. GST activity was assessed by the reaction between 1-chloro-2,4-dinitrobenzene (CDNB) and glutathione. GR activity was measured by the reduction of glutathione. TrxR activity was determined by the reduction of disulfide bonds in thioredoxin. The activities were recorded at specific wavelengths and time intervals and were expressed as enzyme units.

### 2.3 Metabolomics analysis

For metabolomics analysis, strain PM1 was cultured in a selenite-containing medium to assess its metabolic response to selenite stress. Samples were collected at the exponential growth phase, with and without sodium selenite (50 mM) treatment, with six biological replicates for each group. The cultures were centrifuged at 4 °C to collect cell pellets, which were then rapidly quenched with liquid nitrogen. Metabolites were extracted using a methanol–water solution (4,1, v/v) and processed for untargeted metabolomics analysis using liquid chromatography-mass spectrometry (LC–MS). Metabolite identification and quantification were performed based on retention times and mass-to-charge ratios (m/z) using Progenesis QI (Waters Corporation, Milford, United States). To ensure the accuracy of metabolite quantification, data normalization was performed by normalizing the response intensities of the sample mass spectrometry peaks to the sum of their intensities. This method was applied to correct for any systematic variation arising from sample preparation or instrument instability. Additionally, internal standard peaks were used to correct for variations across samples, with known false-positive peaks and noise removed during data processing. The metabolites were then identified by matching their profiles against databases using the self-compiled Majorbio Database (MJDB) of Majorbio Biotechnology Co., Ltd. (Shanghai, China), and the final data matrix was uploaded to the Majorbio cloud platform^1^ for further analysis.

Significant differences in metabolite levels between selenite-treated and control groups were identified through statistical analysis with the criteria of variable importance in projection (VIP) values (>1) and *p*-value (<0.05). To analyze the metabolic differences between the selenite-treated and control groups, unsupervised principal components analysis (PCA) was performed to visualize overall sample clustering and variation. In addition, supervised partial least squares-discriminant analysis (PLS-DA) was applied to maximize the separation between the groups and identify metabolites contributing to the observed differences using R software (version 4.3.3) with the ropls package (version 1.62). Differential metabolites between the two groups were further analyzed through metabolic enrichment and pathway analysis based on the KEGG database.^2^

### 2.4 Transcriptome analysis

After exposure to 50 mM Na_2_SeO_3_ at the exponential growth phase, 10 mL of culture medium was centrifuged at 4000 r/min for 10 min at 4 °C and the cells were collected. A culture medium without Na_2_SeO_3_ was used as the control. Three biological replicates were used for both the selenite-treated and control groups. Total RNA was extracted from cells using the EZNA Bacterial RNA Kit (Omega, GA, United States) following the manufacturer’s protocol. The quality and concentration of RNA were assessed using the NanoDrop 2000 spectrophotometer (Thermo Scientific, United States) and agarose gel electrophoresis. High-quality RNA (RNA integrity number ≥7.0) was used to construct cDNA libraries with the EX RT Kit (gDNA remover) (ZOMANBIO, ZR108-2, Beijing, China). The libraries were then sequenced on the Illumina NovaSeq platform, generating paired-end reads of 150 bp. Raw reads were subjected to quality control using FastQC, followed by trimming of adapter sequences and removal of low-quality reads using Trimmomatic. Clean reads were mapped to the reference genome (CP104782) using Bowtie2^3^, and gene expression levels were quantified using RSEM (Li and Dewey, 2011). Differential expression analysis was performed using DESeq2 with a false discovery rate (FDR) of less than 0.05, and genes with a |log2 fold change| ≥ 1 were considered differentially expressed genes (DEGs). PCA was performed to assess the overall sample clustering and variation. Functional annotation of DEGs was conducted using Gene Ontology (GO) and Kyoto Encyclopedia of Genes and Genomes (KEGG) databases to identify enriched biological processes and pathways. The raw data generated in the present study have been deposited in the NCBI database under the accession number PRJNA881770.

### 2.5 Quantitative real-time PCR validation

To validate the transcriptomic data, a subset of DEGs was selected for qRT-PCR analysis. First-strand cDNA was synthesized from 1 μg of total RNA using the EZNA Bacterial RNA Kit (Omega, GA, United States) following the manufacturer’s instructions. qRT-PCR was performed in a 20 μL reaction volume using 2 × HQ SYBR qPCR Mix (ZOMANBIO, ZF503, Beijing, China) on a StepOneTM Real-Time PCR system (Applied Biosystems, United States). The gene-specific primers were designed using Primer3 software, and 16S rRNA gene was used as the internal reference control (Supplementary Table S1). The thermal cycling conditions were as follows: 95 °C for 30 s, followed by 40 cycles of 95 °C for 10 s, 60 °C for 30 s, and a melt curve analysis was performed to verify the specificity of the amplifications. Relative gene expression levels were calculated using the 2^(−ΔΔCt) method, and each reaction was performed in triplicate. The expression levels of the target genes were compared to the transcriptome data to ensure consistency.

### 2.6 Statistical analysis

Data were presented as standard deviation ± mean and significance analysis was performed using SPSS software with a significance level of *p* < 0.05. Different letters indicate significant differences. R Studio with R version 4.3.1 was used for statistical analysis of the experimental results. All experiments were performed in triplicate copies.

## 3 Results and discussion

### 3.1 Relevant enzyme activities under different Se(IV) stresses

**Figure 1:**
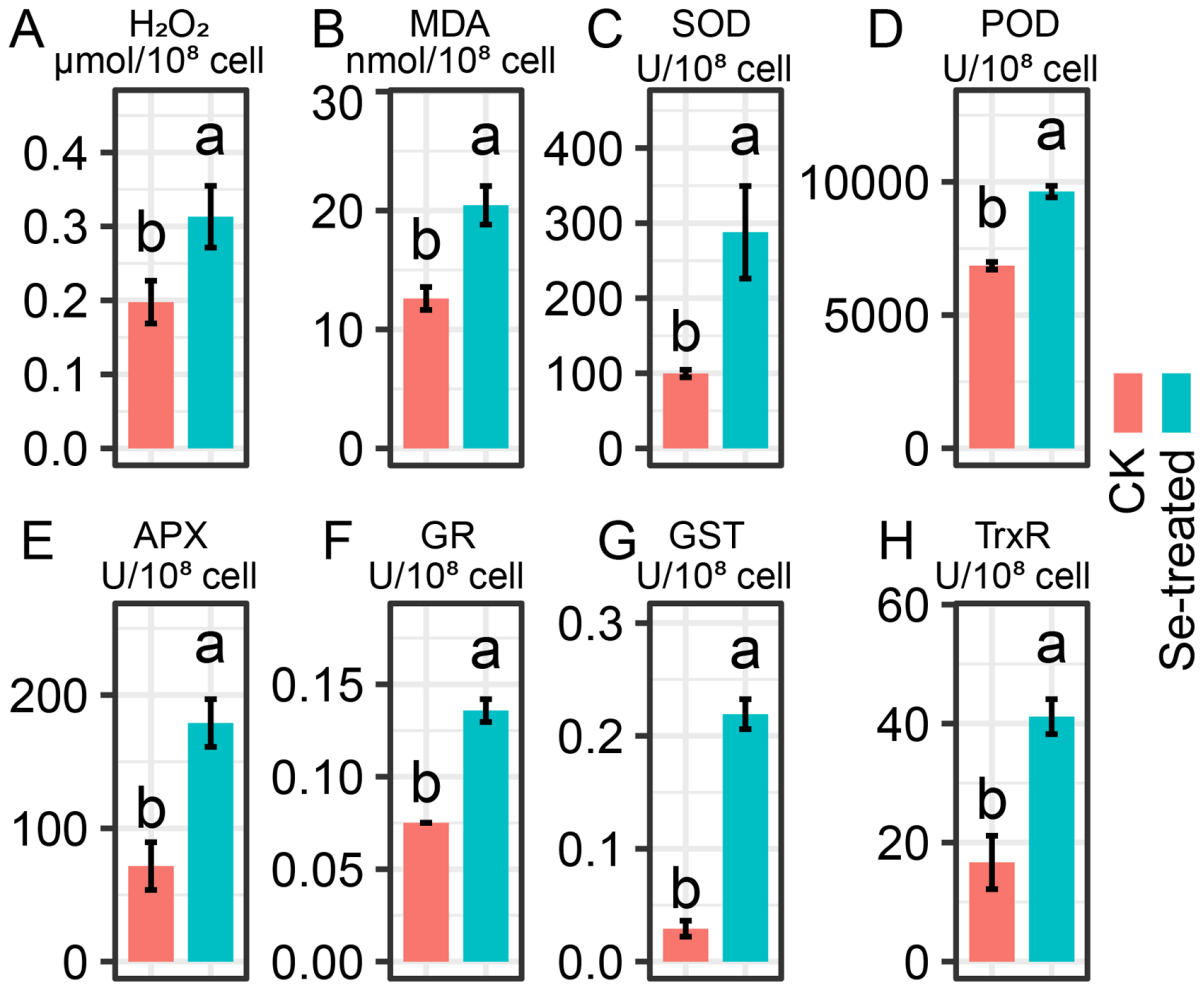
Oxidative stress indicators and antioxidant enzyme responses in *R. qingshengii* PM1 under selenite stress. **(A)** H₂O₂ and **(B)** MDA contents, as well as the activities of **(C)** superoxide dismutase (SOD), **(D)** peroxidase (POD), **(E)** ascorbate peroxidase (APX), **(F)** glutathione reductase (GR), **(G)** glutathione S-transferase (GST), and **(H)** thioredoxin reductase (TrxR), were determined in control (CK) and 50 mM Na₂SeO₃-treated cells. Different letters above the bars indicate significant differences between treatments (*p* < 0.05).

Exposure to Na_2_SeO_3_ induced pronounced oxidative stress in strain PM1, as reflected by significant increases in both H_2_O_2_ and MDA levels (1.6-fold and 1.7-fold higher than the control, respectively), indicating enhanced production of ROS and lipid peroxidation damage (Figures 1A,B). These results suggest that selenium exposure triggers a substantial oxidative challenge within the cells (Chen et al., 2019). To counteract this oxidative challenge, the activities of multiple antioxidant enzymes were markedly upregulated. Specifically, SOD activity increased by ~2.3-fold, suggesting an accelerated dismutation of superoxide radicals into hydrogen peroxide (Figure 1C). Consistently, POD activity rose by ~1.3-fold (Figure 1D), reflecting the enhanced breakdown of H_2_O_2_. In addition, APX activity increased nearly 3-fold, highlighting the role of the ascorbate–glutathione cycle in fine-tuned H_2_O_2_ scavenging under Se stress (Figure 1E). Enzymes directly linked to glutathione homeostasis, including GR (~1.6-fold upregulation) and GST (~7-fold upregulation), were strongly induced (Figures 1F,G), underscoring the importance of glutathione recycling and conjugation in detoxification processes. TrxR activity also rose by ~3-fold, indicating the involvement of the thioredoxin system in maintaining cellular redox balance (Figure 1H). Together, these results demonstrate that strain PM1 mobilizes a coordinated antioxidant defense strategy under selenite stress.

While these upregulations of antioxidant enzymes demonstrate a robust defense mechanism against ROS, other compensatory mechanisms may also contribute to maintaining redox homeostasis and protecting the cell. For example, glutathione and thioredoxin systems are often coupled, and their combined upregulation likely ensures not only ROS detoxification but also maintain protein thiol homeostasis and proper cellular function (Shimizu et al., 2021). These systems may synergize with the enzymatic responses to provide optimal protection against oxidative damage (Li et al., 2025). Thus, while the increased enzyme activities provide significant insight into the cellular response to selenite-induced oxidative stress, further investigations are needed to fully understand the interplay between these enzymatic and non-enzymatic systems in ROS reduction and cellular protection. To explore this further, we conducted transcriptomic and metabolomic analyses.

### 3.2 Se exposure induced differential metabolites in strain PM1

**Figure 2:**
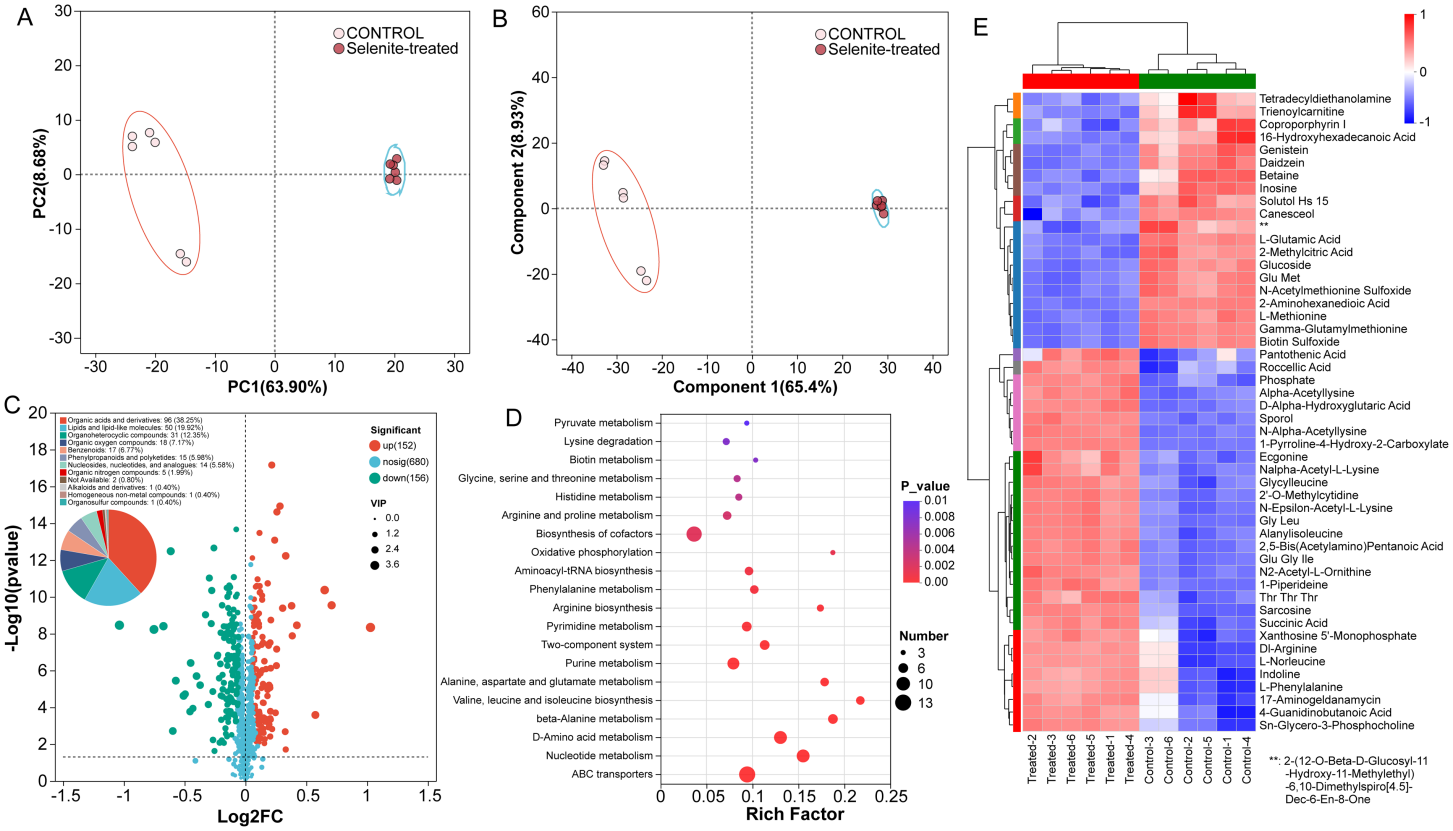
Metabolic profiling of *R. qingshengii* PM1 under selenite exposure. **(A)** Principal component analysis (PCA) of metabolic profiles from control and selenite-treated PM1 samples. **(B)** Partial least squares-discriminant analysis (PLS-DA) of metabolic profiles from control and selenite-treated PM1 samples. **(C)** Volcano plot showing the distribution of differentially expressed metabolites. Red and green dots represent significantly upregulated and downregulated metabolites, respectively, while gray dots indicate non-significant metabolites. The size of the dots reflects the VIP (Variable Importance in Projection) score, and significant metabolites are identified based on log2 fold-change (Log2FC) and *p*-value thresholds. **(D)** KEGG pathway enrichment analysis of the differentially expressed metabolites. The x-axis represents the rich factor (ratio of significant metabolites to total metabolites in each pathway), while the color gradient indicates the *p*-value. The size of the circles corresponds to the number of metabolite enriched in each pathway. **(E)** Hierarchical clustering heatmap of differentially expressed metabolites (DEMs) between control and selenite-treated samples. Each row represents an individual metabolite and each column represents a sample. The color scale indicates the relative abundance of metabolites.

To investigate the metabolic response of strain PM1 under Se stress, LC–MS was used to identify differential metabolites associated with Se exposure. Multivariate PCA and PLS-DA revealed clear separation between the control and Se-treated groups, with significant differences observed in the Se-treated groups (Figures 2A,B), indicating that Se stress significantly alters PM1’s metabolism. Through LC–MS analysis, a total of 988 compounds were identified and semi-quantified, with 560 detected in positive ion mode and 428 in negative ion mode (Supplementary Table S2). These results indicated that significant metabolic changes occurred in strain PM1 under Se stress. A total of 308 differential metabolites (DEMs, VIP > 1 and *p* < 0.05) were identified in strain PM1 when exposed to Se stress, of which 152 were upregulated and 156 were downregulated (Figure 2C; Supplementary Table S3). These DEMs mainly included bioactive compounds, organic acids, lipids, secondary metabolites and organoheterocyclic compounds (Figure 2C), suggesting that the metabolic response of PM1 to Se stress is complex and multifaceted. Metabolic enriched KEGG pathway analysis showed that pyruvate metabolism, arginine and proline metabolism, and phenylalanine metabolism were among the most significantly affected pathways in selenium-exposed conditions, with a large proportion of metabolites showing differential expression (Figure 2D). The results of the clustering analysis of differential metabolites (Figure 2E) showed that the expression modules of DEMs were categorized into up-regulated and down-regulated modules (e.g., compared to the control, the levels of pantothenic acid and succinic acid were up-regulated in treated groups).

Among these DEMs, 33 key metabolites with VIP > 2.0 were further selected for detailed analysis (Table 1). Notably, several metabolites were upregulated, including prostaglandin D3, which is involved in lipid signaling (Fabre et al., 2025), suggesting a role in mediating the stress response to Se exposure (Shi et al., 2025). Tert-Butyl 4-(1H-Pyrazolo[3,4-D]Pyrimidin-4-Yl)Piperazine-1-Carboxylate and 2-Aminooctanedioic acid, associated with nitrogen and amino acid metabolism (Fischle et al., 2024), respectively, were also increased, suggesting that the strain may be shifting toward processes involved in protein synthesis and repair under Se stress. Additionally, metabolites such as (3R,4R)-3-Amino-1-Hydroxy-4-Methylpyrrolidin-2-One and ovalicin were upregulated, potentially reflecting enhanced defense and antimicrobial activity. Furthermore, ribose-5-phosphate, a metabolite linked to the pentose phosphate pathway, likely contributes to providing reducing power (NADPH) for redox balancing under Se exposure (Wang et al., 2022; Shangguan et al., 2024).

**Table 1:** Differential metabolites in Se-treated strain PM1 (VIP > 2 and *p*-value <0.05).

Metabolites	VIP	*P*-value	Log2 FC
Physangulide	3.5898	3.47E-09	−1.037
Prostaglandin D3	3.5837	4.61E-09	1.0276
Enhydrin	3.2921	5.85E-09	−0.7528
Hydroxypelenolide	3.0259	4.29E-11	0.6511
Sebacic Acid	2.8628	3.91E-09	−0.6739
Tert-Butyl 4-(1H-Pyrazolo[3,4-D]Pyrimidin-4-Yl)Piperazine-1-Carboxylate	2.8271	2.89E-10	0.7084
Afn-1252	2.7097	3.32E-13	−0.6151
Methacycline	2.6045	2.37E-05	−0.5082
2-Aminooctanedioic Acid	2.5342	0.00026	0.5745
Olradipine	2.5284	3.57E-06	−0.573
Aklavin	2.4432	0.00194	−0.5971
O-Cresol	2.4358	3.92E-07	−0.4505
(3R,4R)-3-Amino-1-Hydroxy-4-Methylpyrrolidin-2-One	2.4163	3.50E-09	0.4221
9,10-Dihydroxystearic Acid	2.3924	2.03E-06	−0.4024
5-Hydroxyhexanoylglycine	2.3463	5.91E-13	0.3305
Ovalicin	2.2773	3.05E-10	0.3796
Carisoprodol	2.2723	6.22E-06	−0.3725
3-Methoxybenzenepropanoic Acid	2.2319	1.89E-05	−0.4961
Glutarylglycine	2.2075	1.19E-15	0.2837
2,3-Dihydro-2-(3,4-Dihydroxyphenyl)-3-Carboxy-1,4-Benzodioxin-6-Acrylic Acid	2.1917	0.000171	−0.4558
Ribose-5-Phosphate	2.1727	4.12E-10	0.3059
Walleminone	2.1281	0.000114	−0.4328
10-Hydroxydecanoic Acid	2.1338	1.31E-08	0.3844
Tetrahydrodipicolinate	2.1035	2.43E-15	0.2583
Ergothioneine	2.0964	7.03E-05	−0.3051
3-Hydroxy-2-Methyl-4-Pyrone Sulfate	2.0718	9.43E-10	−0.3285
Phenolphthalein	2.0616	4.50E-11	−0.2768
S-(2-Hydroxy-3-Buten-1-Yl)Glutathione	2.0531	9.48E-12	−0.2976
Diglykokoll	2.0524	2.86E-09	−0.2824
1-Pyrroline-4-Hydroxy-2-Carboxylate	2.0291	6.93E-18	0.2162
Sedoheptulose 7-Phosphate	2.0255	5.49E-07	0.2542
2-Isopropylmalic Acid	2.0172	1.67E-07	−0.2309
Hypoglycin B	1.9954	8.32E-14	0.2399

On the other hand, metabolites involved in secondary metabolism and lipid biosynthesis, such as physangulide, enhydrin, and sebacic acid, were significantly downregulated, suggesting a reprogramming of metabolic pathways away from these processes under Se stress. Other downregulated metabolites, including hydroxypelenolide and methacycline, also reflect changes in the strain’s metabolic focus, with a reduction in defense-related pathways. Overall, Se exposure induced a complex metabolic reprogramming in strain PM1, emphasizing alterations in stress response, amino acid metabolism, and energy production while reducing secondary metabolism and lipid biosynthesis.

### 3.3 Se exposure induced differential expression genes in strain PM1

To investigate the DEGs of strain PM1 with or without Se, PM1 cells were exposed to 50 mM selenite for 2 days, followed by RNA-seq. A total of six samples were sequenced on the Illumina platform, generating 20.18 Gb of raw data (Supplementary Table S4). After removing residual rRNA and low-quality reads, 18.61 Gb of clean data were obtained, with each sample yielding more than 2.97 Gb. The percentage of Q20 bases exceeded 98.32%, and Q30 bases accounted for more than 94.68% (Supplementary Table S4), indicating that the sequencing data were of high quality and suitable for further analysis. The transcriptome of PM1 strain was predicted to contain 5,916 CDSs with a GC content of 62.48%, of which the majority were successfully annotated in the NR, Swiss-Prot, Pfam, COG, GO, and KEGG databases (46.74–99.97%) (Supplementary Table S5).

**Figure 3:**
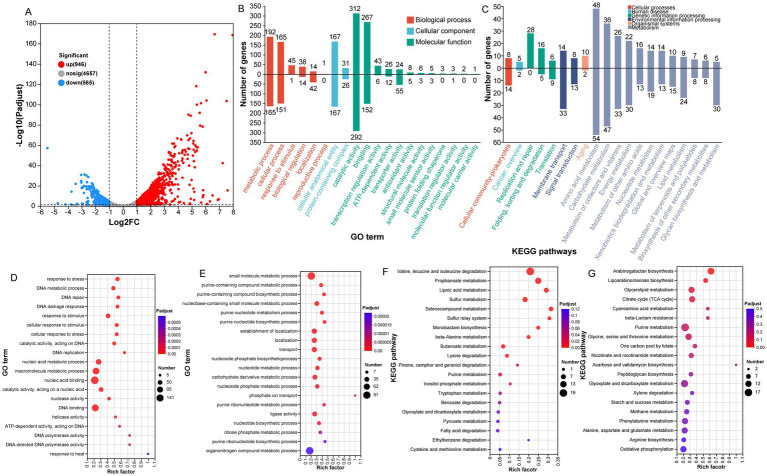
Differential gene expression and functional enrichment analysis in *R. qingshengii* PM1 under selenite exposure. **(A)** Volcano plot of differentially expressed genes (DEGs) between *R. qingshengii* PM1 exposed to 50 mM selenite and the control group. Red dots represent upregulated genes, blue dots represent downregulated genes. The selection criteria for DEGs were log2 fold change | > 1.0| and *p* < 0.05. **(B)** Gene Ontology (GO) annotation of the identified DEGs. **(C)** KEGG annotation of DEGs. **(D)** GO enrichment analysis of the top 20 GO terms among the upregulated DEGs. **(E)** GO enrichment analysis of the top 20 GO terms among the downregulated DEGs. **(F)** KEGG pathway enrichment analysis for upregulated DEGs. **(G)** KEGG pathway enrichment analysis for downregulated DEGs.

Based on |log2Ratio| ≥ 1 (*p* < 0.05), a total of 1,511 DEGs were identified in PM1 after exposure to 50 mM selenite (Figure 3A), among which 946 genes were downregulated and 565 genes were upregulated (Supplementary Table S6). The expression data obtained from RNA-seq were validated using qRT-PCR. The expression of 10 genes randomly selected from the DEGs, was detected using qRT-PCR and compared with the RNA-seq results. The gene expression trends obtained by qRT-PCR were similar to those observed in RNA-seq (Supplementary Figure S1), indicating that the gene expression data obtained from RNA-seq were accurate and reliable.

Based on GO functional analysis, the DEGs were categorized into three main GO domains: biological processes, molecular functions, and cellular components (Figure 3B). Within the biological process domain, the DEGs were significantly enriched in metabolic process (20.30%) and cellular process (17.44%). In the molecular function domain, genes associated with catalytic activity (32.98) and binding activity (28.22) were predominantly represented. For cellular components, the DEGs were primarily related to cellular anatomical entity (17.65%). GO enrichment of the top 20 GO terms showed that the functional proteins involved in the response to stress (GO:0006950), DNA metabolic process (GO:0006259), DNA repair (GO:0015419), DNA damage response (GO:0005524), and response to stimulus (GO:0050896) were enhanced fundamentally in the presence of selenite (Figure 3D). In contrast, genes assigned to small molecule metabolic process (GO:0044281), purine nucleotide biosynthetic process (GO:0006164), purine nucleotide metabolic process (GO:0006163), and nucleobase-containing small molecule metabolic process (GO:0015980), were significantly downregulated (Figure 3E).

In parallel, KEGG pathway analysis was performed to map the DEGs to known metabolic and signaling pathways. The analysis revealed that the DEGs were significantly enriched in amino acid and carbohydrate metabolisms (Figure 3C). Notably, the pathways involved in valine, leucine and isoleucine degradation (map00280), propanoate metabolism (map00640), lipoic acid metabolism (map00785), sulfur metabolism (map00920), selenocompound metabolism (map00450), and sulfur relay system (map04122) was prominently enriched in the upregulated DEGs (Figure 3F). On the other hand, the downregulated DEGs were primarily enriched in pathways associated with arabinogalactan biosynthesis (map00572), lipoarabinomannan biosynthesis (map00571), glycerolipid metabolism (map00561), and citrate cycle (TCA cycle) (map00020) (Figure 3G).

### 3.4 Association analysis of transcriptome and metabolome in PM1 response to Se

**Figure 4:**
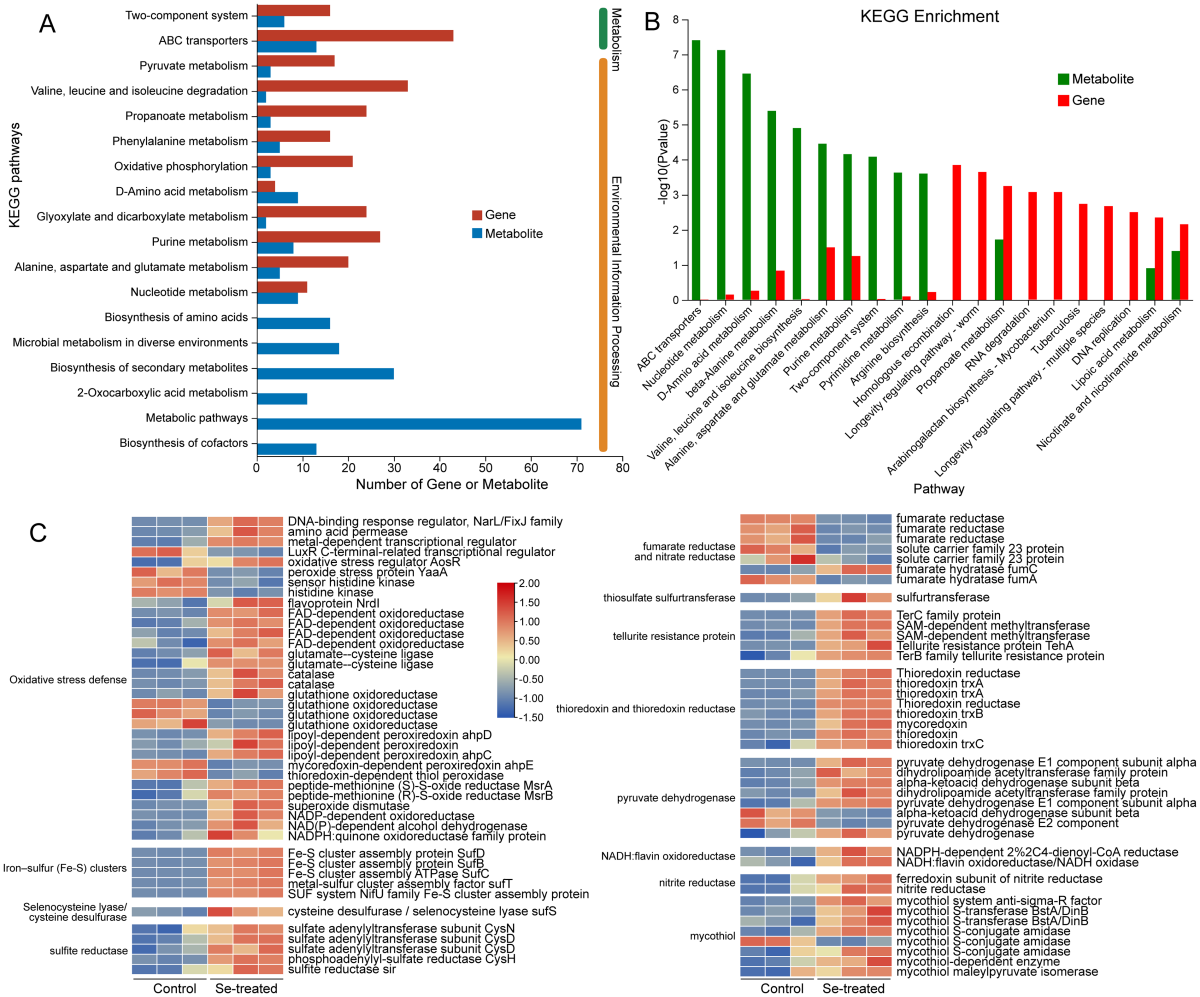
KEGG pathway enrichment analysis of genes and metabolites in PM1 response to selenite stress. **(A)** The number of genes (in red) and metabolites (in blue) involved in each enriched KEGG pathway. **(B)** The significance of the KEGG pathways based on the *p*-value, with −log10 (*p*-value) on the y-axis. The higher the bar, the more significant the pathway is. Green bars represent the enriched metabolites, and red bars represent the enriched genes in the respective pathways. **(C)** Heatmap of different expressed genes changes in PM1 response to selenite stress.

An association analysis of the transcriptome and metabolome was performed to gain a comprehensive understanding of the regulatory mechanisms involved in PM1’s response to selenite stress. A large number of DEGs were found to be strongly correlated with different DEMs, particularly in pathways related to the two-component system, ABC transporters, and amino acid metabolism (Figure 4A). The KEGG enrichment analysis highlighted significant changes in metabolic pathways such as valine, leucine, and isoleucine biosynthesis, as well as oxidative phosphorylation (Figure 4B). These changes were driven by the respective genes involved in these pathways, suggesting a reconfiguration of energy metabolism and amino acid utilization under stress conditions. The integration of transcriptomic and metabolomic data also revealed that pathways like nucleotide metabolism and D-amino acid metabolism were also notably enriched, indicating a potential adaptive mechanism to mitigate selenite-induced oxidative stress. These findings provide valuable insights into the complex interplay between gene expression and metabolic adjustments in PM1 during selenite stress, offering potential targets for further functional studies and biotechnological applications.

### 3.5 Main genes or metabolites potential participating in selenite reduction and SeNRs biosynthesis

Selenium, while classified as a metalloid, primarily exerts stress effects on microbial cells at high concentrations due to the high reactivity of selenium-oxygen associations, particularly under aerobic conditions (Khanna et al., 2023). This stress can induce the production of reactive oxygen species (ROS), which can damage cell membranes or DNA. In response to this oxidative stress and maintain redox homeostasis, strain PM1 activated various antioxidant defense mechanisms, similar to the responses observed in other selenite-resistant bacteria, such as *Proteus* sp. (Wang et al., 2022) and *Providencia rettgeri* (Huang et al., 2021). In strain PM1, genes involved in oxidative stress defense (Figure 4C; Supplementary Table S6), such as *nrdI* (ribonucleoside-diphosphate reductase) (1.28-fold), *fpr* (FAD-dependent oxidoreductase) (4.93, 2.42, 2.11, 2.47, 1.38 and 1.37-fold), and multiple antioxidant proteins (Figure 4C; Supplementary Table S6), including *gshA* (glutathione synthetase) (1.91 and 1.55-fold), *ahpC/ahpD* (alkyl hydroperoxide reductase) (2.61 and 2.92-fold), *katE* (catalase) (2.11 and 1.69-fold), *gor* (glutathione oxidoreductase) (1.69-fold), *ahp* (peroxiredoxin) (2.61 and 1.37-fold), *msr* (methionine sulfoxide reductase) (1.44 and 2.44-fold), *sodB* (superoxide dismutase) (2.47-fold), *qor* (NADPH:quinone reductase) (2.40, 2.08 and 1.08-fold), were significantly upregulated in response to selenite exposure (Khanna et al., 2023; Ge et al., 2024). However, not all antioxidant proteins exhibited increased expression after selenium treatment, indicating a selective activation of antioxidant pathways (Song et al., 2017). Glutarylglycine (VIP 2.2075) also showed an increase (Table 1), reinforcing the role of amino acid and glutathione conjugation in the microbial response to selenium stress. Conjoint transcriptome and metabolome analysis showed that the glutathione metabolic pathway was over-represented in both DEGs and DEMs.

In parallel, the upregulation of ribose-5-phosphate (VIP 2.17) (Table 1), a key intermediate in the pentose phosphate pathway (PPP), likely contributes to increased NADPH production, and the expression of multiple related genes, including *6pgl* (6-phosphogluconolactonase), *gnd* (glucose-6-phosphate dehydrogenase), *zwf* (glucose-6-phosphate 1-dehydrogenase), *talA* (transaldolase), and *tktA* (transketolase), may further reinforce this process (Wang et al., 2025). NADPH serves as a reducing agent critical for combating ROS and maintaining redox homeostasis (Xiao et al., 2018). The role of the PPP in selenium metabolism mirrors findings in *Proteus* sp. where upregulation of the PPP under selenium stress contributed to the replenishment of NADPH and assisted in the reduction of selenite (Wang et al., 2022).

The role of lipid metabolism in selenium stress was further evidenced by the significant changes in lipid-related metabolites, including prostaglandin D3 (VIP 3.58) and physangulide (VIP 3.59) (Table 1). These metabolites, although not directly involved in selenite reduction, suggest that selenium exposure induces widespread disruptions in cell lipid metabolism, possibly affecting membrane stability and integrity (Bai et al., 2019). Lipid remodeling, such as changes in fatty acid composition, has been observed in other heavy metal-resistant bacteria, where it aids in membrane fluidity and protection against oxidative damage (Wu et al., 2022). The change in 9,10-Dihydroxystearic Acid (VIP 2.39) further suggested that strain PM1 is employing lipid modification as part of its adaptive strategy to selenium stress. Similar mechanisms have been observed other studies, where changes in membrane lipids were associated with enhanced resistance to metal stress (Ghosh et al., 2018).

Iron–sulfur (Fe-S) clusters, essential for maintaining cellular redox balance and metabolic function, are synthesized by the SUF, NIF, and ISC macromolecular machines (Esquilin-Lebron et al., 2021; Pedroletti et al., 2023). The high reactivity of iron and sulfide in cells necessitates tight regulation of iron–sulfur (Fe-S) cluster assembly (Rodrigues et al., 2015). In strain PM1, exposure to selenite resulted in a significant increase in the expression of genes involved in the SUF system, including *sufA*, *sufB*, *sufC*, *sufD*, and *sufT*, with fold changes ranging from 2.92 to 3.54 (Figure 4C; Supplementary Table S6). This suggests that Fe-S clusters may play a crucial role in the reduction of selenite in this strain. Additionally, the upregulation of *iscU* (3.08-fold) further supports the involvement of iron–sulfur cluster assembly systems in selenite reduction in strain PM1. Additionally, selenocysteine lyase activity was detected in several bacteria and can decompose L-selenocysteine into elemental selenium (Seale, 2019). Selenocysteine lyase/cysteine desulfurase (*sufS*) was upregulated in strain PM1 (1.79-fold) when selenite was added (Figure 4C; Supplementary Table S6). However, *SufS* does not appear to be important for selenium metabolism in *Escherichia coli* (Mihara et al., 2002).

Interestingly, strain PM1 exhibited a downregulation of genes associated with fumarate and nitrate reductases (Supplementary Table S6), which are known to participate in selenite reduction in *Enterobacter cloacae* Z0206 and *Rahnella aquatilis* HX2 (Song et al., 2017; Li et al., 2024). Moreover, fumarate hydratase, which participates in the tricarboxylic acid cycle, exhibited a mixed response to selenite stress, with both upregulation and downregulation observed (1.22 and −1.18-fold), in contrast to previous studies where this enzyme was repressed under selenite stress (Gómez-Gómez et al., 2019). This suggests that strain PM1 relies on alternative pathways, possibly linked to Fe-S clusters, for reducing selenite, rather than the nitrate reductase systems utilized by these other strains. This divergence in the utilization of reductases highlights the versatility of bacterial selenite reduction strategies, depending on environmental conditions and genetic adaptations.

In response to Na_2_SeO_3_ stress, multiple transporters were significantly upregulated in PM1, including ABC transporters, MFS transporters, sugar transporters, and ion transporters (Supplementary Table S6). However, the key high-affinity phosphate transport system Pst (*pstS*, *pstA*, *pstB*, *pstC*) was markedly downregulated (Supplementary Table S6), suggesting that PM1 reduces phosphate uptake to regulate intracellular phosphate homeostasis. In addition, the sulfur ABC transporter complex CysAWTP is also implicated in selenium uptake and metabolism (Tugarova and Kamnev, 2017). In *E. coli*, Se(IV) uptake is mediated by the sulfate ABC transporter complex encoded by the *cysAWTP* operon, and evidence suggests that *E. coli* possesses an independent Se(IV) transport system that facilitates its intracellular translocation (Turner et al., 1998; Rosen and Liu, 2009). Although a complete cysAWTP operon was identified in the PM1 genome (Wang et al., 2025), its transcriptional levels were not significantly altered under selenite stress. However, genes associated with sulfite reduction, including *cysN*, *cysD*, *cysH* (Figure 4C; Supplementary Table S6), and *sir*, were markedly upregulated, suggesting that while cysAWTP may still play a role in selenium reduction, PM1 appears to predominantly rely on the sulfate/sulfite reduction pathway under selenite stress. Metabolomic analysis further corroborated this observation: metabolites directly associated with CysAWTP transport were undetected, whereas intermediates of the sulfate reduction pathway accumulated significantly, accompanied by elevated levels of reducing cofactors such as NADPH and glutathione, indicating that PM1 detoxifies selenite mainly through a mechanism coupling sulfate/sulfite reduction with cellular metabolism.

In addition to the role of Fe-S clusters, the enzyme thiosulfate sulfurtransferase (TST), which is involved in both sulfur and selenium metabolism, was also significantly upregulated in strain PM1 (2.08-fold). TST helps in reducing antioxidants such as glutathione and thioredoxin, highlighting its contribution to maintaining cellular redox balance under selenite stress (Kruithof et al., 2020). Genes associated with tellurite resistance protein were also significantly upregulated in strain PM1, including *terB* (1.50-fold), *terC* (4.98-fold), *tehA* (6.51-fold), and *tehB* (2.13 and 2.03-fold). This indicates a potential cross-resistance mechanism between tellurite and selenite stress, as tellurite resistance proteins have been implicated in oxidative stress defense and metal ion detoxification (Ali et al., 2019).

Interestingly, a total of eight genes involved in thioredoxin and thioredoxin reductase were significantly upregulated in strain PM1 under selenite treatment (Figure 4C; Supplementary Table S6). Thioredoxin systems, like those observed in *Saccharomyces cerevisiae* ATCC MYA-2200, are known to reduce selenite to Se^0^ (Kieliszek et al., 2019). Additionally, the expression of pyruvate dehydrogenase complex genes (*pdhA/pdhB/pdhC/pdhR*) significantly increased in strain PM1 in the presence of selenite (Figure 4C; Supplementary Table S6), suggesting an active oxidative decarboxylation pathway contributing to acetyl-CoA and NADH production (Patel and Korotchkina, 2003), as similarly observed in *Proteus* sp. YS02 (Wang et al., 2022).

NADH:flavin oxidoreductase, which plays a role in maintaining redox homeostasis and reducing oxidative stress, was also significantly upregulated in strain PM1, with fold changes of 2.22 and 1.53 (Figure 4C; Supplementary Table S6). This enzyme, which has been reported to be involved in selenite reduction in *Rhizobium selenitireducens* (Hunter, 2014), may contribute to the reduction of selenite in strain PM1 as well, indicating a potential oxidative stress defense mechanism. Additionally, nitrite reductase genes (*nrfA*), which are also upregulated (1.97 and 1.47-fold) (Figure 4C; Supplementary Table S6), further suggest a broader role for redox-active enzymes in selenite reduction in strain PM1.

Additionally, it is crucial to note that selenium-induced oxidative stress effects are not solely due to its metalloid nature but are more complex, involving its interactions in nanostructures and its behavior under different conditions. Furthermore, *Rhodococcus* species are known to produce mycothiol (MSH) in addition to glutathione, which play a significant role in managing oxidative stress (Elsayed et al., 2017). MSH offers enhanced redox stability and serves as a cysteine reservoir, being less prone to auto-oxidation than GSH (Newton et al., 2008). A total of nine mycothiol-related genes, including mycothiol system anti-sigma-R factor [involved in the biosynthesis of mycothiol (serves as an antioxidant) (Wang et al., 2024), 2.90], mycothiol S-conjugate amidase (1.87 and 1.12), mycothiol S-transferase BstA (2.05 and 1.43), mycothiol maleylpyruvate isomerase (1.58 and 1.16), were detected in PM1 with significantly upregulated (Supplementary Table S7), suggesting that MSH is probably involved in detoxification and redox-buffering reactions under selenite treatment. Similar findings are reported in other *R. qingshengii* strains (Aggarwal et al., 2011; Zumsteg et al., 2023).

### 3.6 Potential mechanism dominating selenite reduction in strain PM1

**Figure 5:**
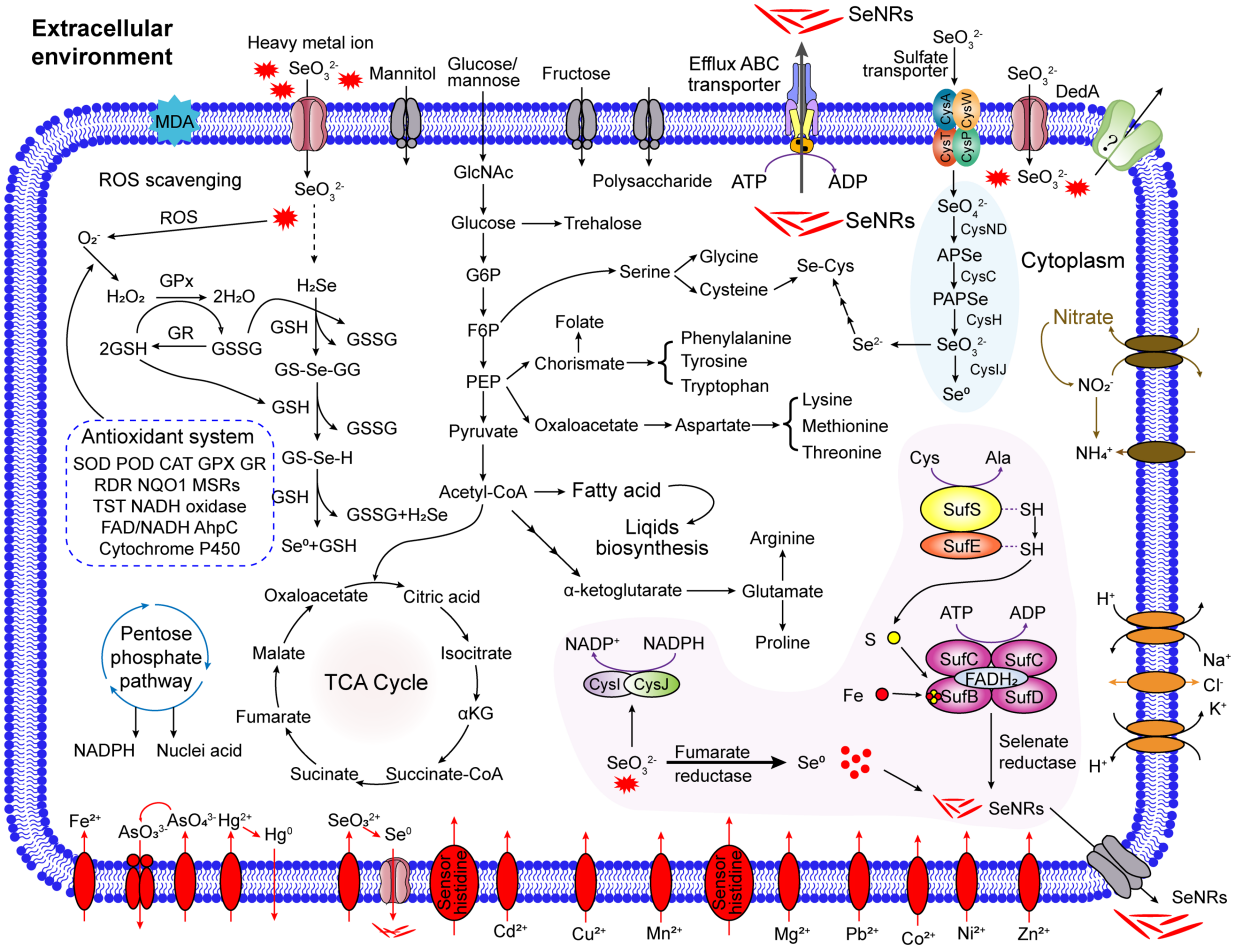
The multiple potential metabolic pathways of selenite in *R. qingshengii* PM1. The sulfur ABC transporter complex (CysAWTP) facilitates the uptake of Se(IV) into the cytoplasm, where NADPH or NADH generated via the pentose phosphate pathway is used by the sulfite reductase complex (CysNDHIJ) to reduce Se(IV) to elemental selenium (Se⁰). Multiple reductases, including glutathione reductase, thioredoxin reductase, and NADH oxidoreductase, contribute to this reduction process. Antioxidant proteins and Fe-S cluster biosynthesis genes (SufBCDSE) help mitigate oxidative stress and enhance selenium reduction. Se⁰ produced in the cytoplasm is likely released into the extracellular space, while non-enzymatic reductions involving agents like iron siderophores, glutathione, and sulfide may also play a role. Upregulation of detoxification genes, such as those related to tellurite resistance, suggests a broad-spectrum stress response in PM1, offering resilience in metal-contaminated environments.

In *R. qingshengii* PM1, selenite reduction appears to be governed by a combination of metabolic pathways (Figure 5). First, the sulfur ABC transporter complex CysAWTP likely facilitates the uptake of Se(IV) into the cytoplasm. Once inside the cell, NADPH or NADH generated by metabolic process could be directed to the sulfite reductase complex (CysNDHIJ), which would act as an electron donor for the reduction of Se(IV) to Se^0^. In addition, selenite reduction might involve multiple reductases, such as glutathione reductase, thioredoxin reductase, nitrite reductase, NADH oxidoreductase, contributing to the reduction of Se(IV) to Se^0^. The upregulation of antioxidant proteins helps to mitigate oxidative stress caused by selenite, while the activation of Fe-S cluster biosynthesis genes (*SufBCDSE*) enhances the cell’s ability to efficiently reduce selenium. The Se^0^ formed in the cytoplasm may be released into the extracellular space. However, further experimental validation is required to clarify these potential mechanisms and their contribution to selenite reduction in *R. qingshengii* PM1. The upregulation of specific detoxification genes, such as those involved in tellurite resistance, further suggests a broad-spectrum stress response in strain PM1, likely conferring an advantage in environments contaminated with multiple toxic metals.

## 4 Conclusion

This study demonstrates that *R. qingshengii* PM1 exhibits exceptional tolerance and reduction capacity toward selenite, effectively converting toxic Se(IV) into elemental selenium. Integrative omics analyses revealed that selenite stress activates a complex regulatory network, including the sulfite reductase system, Fe–S cluster biosynthesis, glutathione-mediated redox balance, and lipid remodeling. These coordinated responses facilitate cellular detoxification and the biosynthesis of selenium nanostructures. Importantly, the findings establish a molecular framework for understanding microbial selenium reduction and position PM1 as a valuable biological resource for the remediation of selenium-contaminated sites.

## Data Availability

The datasets presented in this study can be found in online repositories. The names of the repository/repositories and accession number(s) can be found in the article/Supplementary material.

## Footnotes

^1^ www.majorbio.com

^2^ http://www.genome.jp/kegg/

^3^ http://bowtie-bio.sourceforge.net/bowtie2/index.shtml
