# Supplementary material for: Multi-omics analysis reveals the mechanism of selenite reduction by Rhodococcus qingshengii strain isolated from selenium-rich mine
Source: Front Microbiol. 2025 Nov 14;16:1712891. doi: 10.3389/fmicb.2025.1712891 (PMC12660274; doi:10.3389/fmicb.2025.1712891)
Supplement: Supplementary file 2 [file Data_Sheet_1.docx]

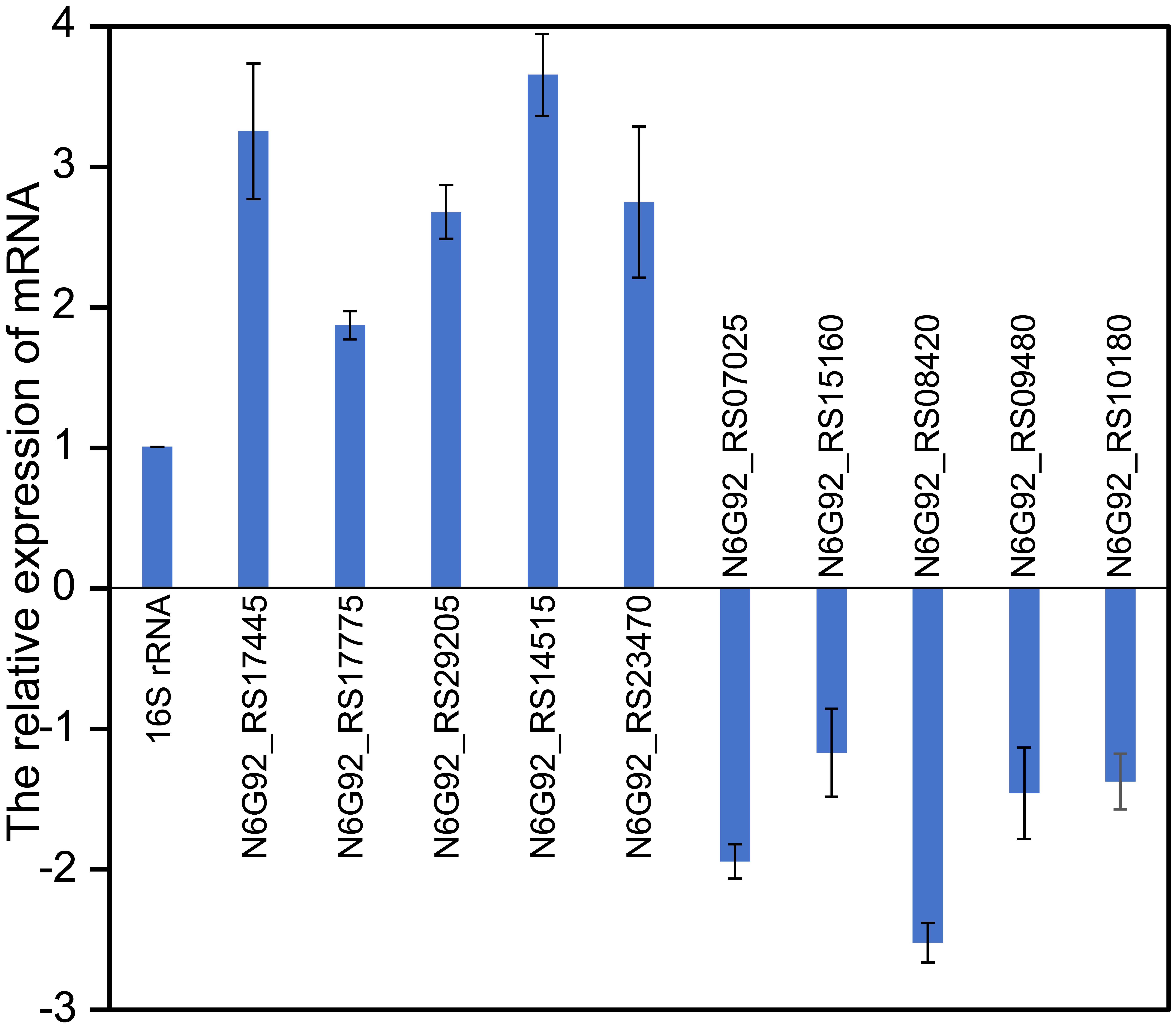


**Fig. S1** Quantitative real-time PCR validation of differentially expressed genes in the *R. qingshengii PM1* transcriptome. All reactions were performed in biological triplicate, and the error bars represent the standard deviations. The corresponding gene names for the gene IDs can be found in Table S2.
